# Volatile Solid Additive‐Assisted Sequential Deposition Enables 18.42% Efficiency in Organic Solar Cells

**DOI:** 10.1002/advs.202105347

**Published:** 2022-01-24

**Authors:** Jianqiang Qin, Qianguang Yang, Jiyeon Oh, Shanshan Chen, George Omololu Odunmbaku, Nabonswendé Aïda Nadège Ouedraogo, Changduk Yang, Kuan Sun, Shirong Lu

**Affiliations:** ^1^ MOE Key Laboratory of Low‐Grade Energy Utilization Technologies and Systems School of Energy & Power Engineering Chongqing University Chongqing 400044 P. R. China; ^2^ Chongqing Institute of Green and Intelligent Technology Chinese Academy of Sciences Chongqing 400714 P. R. China; ^3^ Department of Energy Engineering School of Energy and Chemical Engineering Perovtronics Research Center Low Dimensional Carbon Materials Center Ulsan National Institute of Science and Technology (UNIST) Ulsan 44919 Republic of Korea

**Keywords:** morphology optimization, organic solar cells, sequential deposition, solid additive

## Abstract

Morphology optimization of active layer plays a critical role in improving the performance of organic solar cells (OSCs). In this work, a volatile solid additive‐assisted sequential deposition (SD) strategy is reported to regulate the molecular order and phase separation in solid state. The OSC adopts polymer donor D18‐Cl and acceptor N3 as active layer, as well as 1,4‐diiodobenzene (DIB) as volatile additive. Compared to the D18‐Cl:N3 (one‐time deposition of mixture) and D18‐Cl/N3 (SD) platforms, the D18‐Cl/N3(DIB) device based on DIB‐assisted SD method exhibits a finer phase separation with greatly enhanced molecular crystallinity. The optimal morphology delivers superior charge transport and extraction, offering a champion power conversion efficiency of 18.42% with significantly enhanced short‐circuit current density (*J*_sc_) of 27.18 mA cm^−2^ and fill factor of 78.8%. This is one of the best performances in binary SD OSCs to date. Angle‐dependent grazing‐incidence wide‐angle X‐ray scattering technique effectively reveals the vertical phase separation and molecular crystallinity of the active layer. This work demonstrates the combination of volatile solid additive and sequential deposition is an effective method to develop high‐performance OSCs.

## Graphical abstract

High‐performance organic solar cells (OSCs) based on D18‐Cl and N3 are fabricated by combining volatile solid additive 1,4‐diiodobenzene (DIB) with sequential deposition (SD) strategy. The formed D18‐Cl/N3(DIB) film exhibits favorable phase separation and enhanced crystallinity. The optimized morphology enables a remarkable power conversion efficiency of 18.42%, which is one of the highest performances in binary SD OSCs to date.

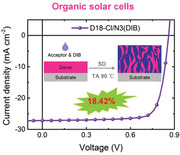

## 1 Introduction

With the aggravation of energy shortage and environmental pollution, it is vital to develop new technology to harvest renewable energy. Organic solar cell (OSC) is a promising candidate due to its merits of lightweight, low cost, flexibility, and tunable optoelectronic properties.^[^ ^1^ ^]^ With the emergence of Y6‐series acceptors, the state‐of‐the‐art bulk heterojunction (BHJ) OSCs have obtained high power conversion efficiency (PCE) over 18%.^[^ ^2^ ^]^ Morphology optimization of the active layer plays a vital role in improving the performance of OSCs. The ideal morphology for the active layer should possess appropriate phase separation, high molecular crystallinity, and interdiffusion between donor and acceptor in the vertical direction.^[^ ^3^ ^]^ To obtain such an ideal morphology, various strategies have been reported, including thermal annealing (TA),^[^ ^4^ ^]^ solvent vapor annealing,^[^ ^5^ ^]^ solvent additive,^[^ ^6^ ^]^ and solid additive,^[^ ^7^ ^]^ etc. The above‐mentioned strategies are mainly coupled with one‐time deposition (OTD) of donor/acceptor mixed solution.

Recently, sequential deposition (SD) method has attracted enormous attention since it has a positive effect on regulating vertical phase distribution.^[^ ^8^ ^]^ In addition, sequentially depositing donor and acceptor layer via the SD method makes it possible to regulate donor and acceptor individually, thus eliminating complex interactions between donor and acceptor. For example, Jen et al. fabricated OSCs with a pseudo‐bilayer (PB) architecture by using the SD method. Compared with the BHJ films produced via OTD, PB films by SD showed enhanced crystallinity and longer exciton diffusion length, which facilitated exciton dissociation and charge transport. Finally, the PB OSCs showed a higher PCE of 17.42% than the BHJ OSCs (16.44%).^[^ ^9^ ^]^ Meanwhile, Chen et al. fabricated high‐performance OSCs by combining SD method and ternary blend strategy. After introducing the third component, the ideal vertical phase distribution was formed. Consequently, the SD‐processed ternary OSCs yielded a record PCE of 18.16%.^[^ ^10^ ^]^ Li et al. prepared the graded active layer in OSCs by combining SD method and nonhalogenated solvent (*o*‐xylene). The resulting graded vertical phase distribution facilitated charge transport, yielding a remarkable PCE of 17.48%. The relevant thick‐film and blade‐coated OSCs also show outstanding PCEs, respectively.^[^ ^11^ ^]^ Very recently, Peng et al. introduced a wax additive to produce an interdigitated heterojunction morphology; an impressive PCE of 18.74%.^[^ ^8b^ ^]^ However, the molecular crystallinity is neglected in all reported SD‐based OSCs, especially for nonfullerene acceptor. So enhancing the crystallinity in SD OSCs is expected to further improve their photovoltaic performances.

Herein, we report an efficient OSC based on the polymer donor D18‐Cl and nonfullerene acceptor N3 system, which is manufactured by combining the SD method with a volatile solid additive 1,4‐diiodobenzene (DIB). Such an OSC device is denoted as D18‐Cl/N3(DIB). To study the synergistic effect of SD method and DIB additive on the morphology of active layer, we fabricate two control cells, i.e., BHJ OSCs (D18‐Cl:N3) and SD OSCs without the additive (D18‐Cl/N3). Morphological studies show that the SD method can construct a BHJ‐like bicontinuous interpenetrating network. With the introduction of DIB volatile solid additive into N3, the D18‐Cl/N3(DIB) film exhibits adjustable phase separation and increased crystallinity. The optimal morphology enables enhanced charge extraction, faster and more balanced charge transfer, and reduced charge recombination, ultimately resulting in superior short‐circuit current density (*J*_sc_) and fill factor (FF). Consequently, the D18‐Cl/N3(DIB) devices delivered a remarkable PCE of 18.42%. The SD method together with volatile additive represents a feasible and effective strategy towards fine tuning the phase separation and molecular crystallinity in OSCs.

## 2 Results and Discussion

**Figure 1:**
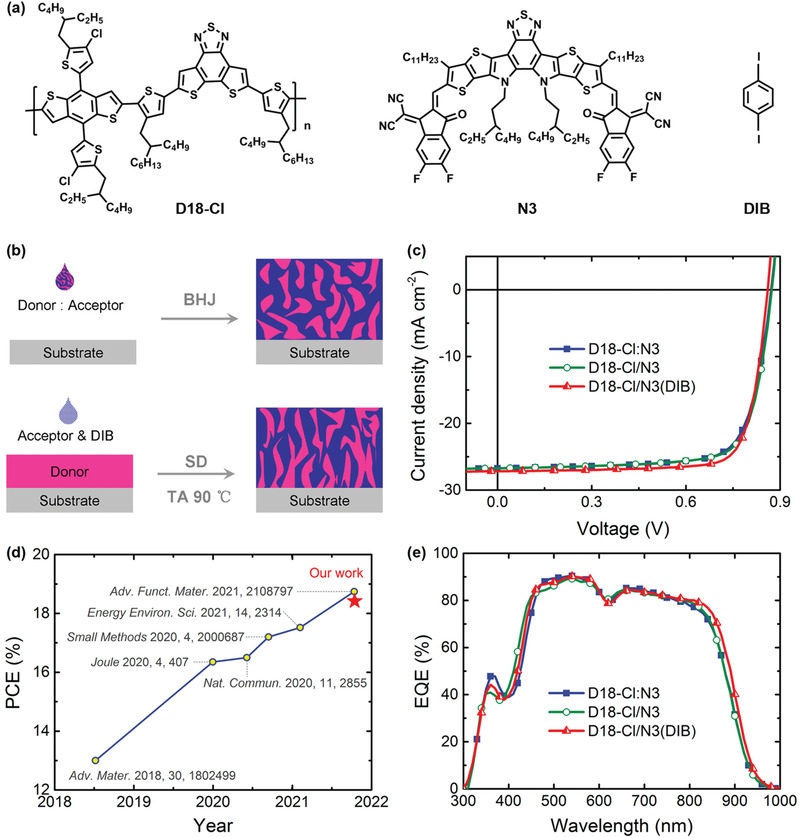
a) Chemical structures of D18‐Cl, N3, and DIB. b) Schematic illustration of the fabrication process for BHJ and SD OSCs. c) *J*–*V* curves for the optimized OSCs. d) Summary of recently reported high‐performance binary SD OSCs. e) EQE spectra of the optimized OSCs.

The chemical structures of polymer donor D18‐Cl, nonfullerene acceptor N3, and volatile solid additive DIB are shown in **Figure** **1**a. The light absorption profiles of the active layers containing the above‐mentioned materials are firstly recorded (Figure S1, Supporting Information). The UV–vis absorption spectrum of the SD D18‐Cl/N3 blend film is practically equivalent to that of BHJ D18‐Cl:N3 film, implying almost identical film thickness and molecular packing. But the absorption spectra of the SD D18‐Cl/N3(DIB) blend film is slightly red‐shifted in the wavelength range of 600–1000 nm. The subtle difference in absorption spectra indicates the DIB additive exhibits a positive effect on improving molecular packing of N3 acceptor.^[^ ^12^ ^]^

Then different techniques are employed to probe whether the DIB additive remains left in the active layer after thermal annealing the as‐cast film. According to thermogravimetry analysis (TGA), DIB exhibits a significant weight loss at 90 °C.^[^ ^7d^ ^]^ To further verify the volatility of DIB, it is spin‐coated on Si wafer and then heated at 90 °C for 5 min. As shown in Figure S2 (Supporting Information), DIB completely vanished after thermal treatment. Furthermore, Fourier transform infrared spectroscopy (FT‐IR) measurements confirm that there is no residual DIB in the blend. The peaks at 463, 795, 990, 1066, and 1460 cm^−1^ assigned to DIB are entirely absent in the annealed D18‐Cl/N3(DIB) blend (Figure S3, Supporting Information).^[^ ^7d^ ^]^ These results together prove DIB is a volatile solid that can be completely removed during TA process.

To evaluate the photovoltaic performance, conventional devices were fabricated with an architecture of ITO/PEDOT:PSS/active layer/PDIN/Ag. As shown in Figure 1b, the active layer was formed from a two‐stage SD process, in which the neat donor polymer D18‐Cl layer was first spin‐coated on the substrate, and then a mixed N3/DIB solution was deposited subsequently. The resultant film was thermally annealed at 90 °C to remove the DIB additive and to fine‐tune the morphology. The current density versus voltage (*J*–*V*) curves are shown in Figure 1c and the corresponding photovoltaic parameters are listed in **Table** **1**. The conventional BHJ OSC, D18‐Cl:N3, shows a PCE of 17.49% with a *J*_sc_ of 26.74 mA cm^−2^ and a FF of 75.1%. As for the SD OSCs, the detailed optimization procedures are summarized in Tables S1 and S2 (Supporting Information). Of note, the optimum thicknesses of 65 and 110 nm were obtained for the donor and active layer in D18‐Cl/N3, respectively. The optimized SD OSC shows a slightly higher PCE of 17.74% and FF of 76.0%. By incorporating adequate amount of DIB additive into acceptor N3 solution (Table S3, Supporting Information), a champion PCE of 18.42% with enhanced *J*_sc_ of 27.18 mA cm^−2^ and FF of 78.8% was achieved. It is worth noting that 18.42% efficiency is one of the few examples with PCEs over 18% in binary SD‐based OSCs to date (Figure 1d; Table S4, Supporting Information).

**Table 1:** Photovoltaic Parameters of the Optimized BHJ and SD OSCs

Active layer	*V_oc_* [V]	*J_sc_* [mA cm^−2^]	FF [%]	PCE [%]^a)^
D18‐Cl:N3	0.871	26.74 (26.23)^b)^	75.1	17.49 (17.23 ± 0.15)
D18‐Cl/N3	0.873	26.74 (26.33)	76.0	17.74 (17.50 ± 0.17)
D18‐Cl/N3(DIB)	0.860	27.18 (26.81)	78.8	18.42 (18.20 ± 0.15)

^a)^ Average values and standard deviation were obtained from 16 individual devices;

^b)^ The integrated current density values calculated from EQE spectra.

Figure 1e presents the external quantum efficiency (EQE) spectra of the three representative devices. The corresponding integrated current density values are summarized in Table 1, which are in good agreement with *J*–*V* curve results. The D18‐Cl/N3(DIB) OSCs exhibit a broader EQE response compared to those without DIB treatment, which results in the highest *J*_sc_ value and consists with the absorption spectra previously mentioned.

**Figure 2:**
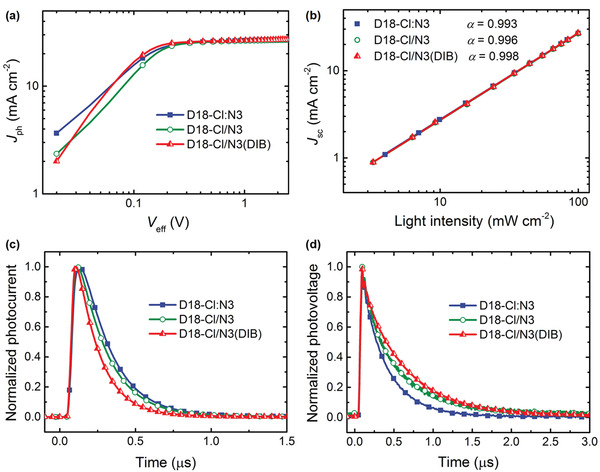
a) *J*_ph_ versus *V*_eff_ curves of the devices. b) *J*_sc_ versus light intensity curves of the devices. c) TPC curves of the devices. d) TPV curves of the devices.

To understand the synergistic effect of the SD method and volatile solid additive DIB on photovoltaic properties, we investigated the exciton dissociation, charge transport, and recombination process in all three systems. The photocurrent density (*J*_ph_) versus effective voltage (*V*_eff_) curves were plotted to probe the exciton dissociation and charge collection process (**Figure** **2**a; Table S5, Supporting Information). *J*_ph_ is determined by *J*_ph_ = *J*_L_ – *J*_D_, where *J*_L_ and *J*_D_ are the current density of the devices under illumination and in dark, respectively. *V*_eff_ is calculated by *V*_eff_ = *V*_0_ – *V*_a_, where *V*_0_ and *V*_a_ are the voltage at *J*_ph_ = 0 and the applied voltage, respectively.^[^ ^13^ ^]^ The exciton dissociation probability (*η*_diss_) can be defined by the ratio of *J*_ph_/*J*_sat_ under short‐circuit condition, where *J*_sat_ is the saturation *J*_ph_. And the charge collection probability (*η*_coll_) can be determined by the ratio of *J*_ph_/*J*_sat_ under maximum power output condition.^[^ ^14^ ^]^ Apparently, the D18‐Cl/N3(DIB) device shows a higher *η*_diss_ (98.1%) and *η*_coll_ (90.7%) than that of D18‐Cl:N3 (*η*_diss_ of 97.4% and *η*_coll_ of 86.6%) and D18‐Cl/N3 (*η*_diss_ of 97.7% and *η*_coll_ of 87.6%). The simultaneous enhancement of exciton dissociation and charge collection probability can promote the increment of *J*_sc_ in the D18‐Cl/N3(DIB) device.

To probe the carrier transport process in OSCs, electron mobility (*μ*_e_) and hole mobility (*μ*_h_) were measured via the space charge limited current (SCLC) method and presented in Figure S4 (Supporting Information). The detailed data of charge mobility were summarized in Table S6 (Supporting Information). Notably, D18‐Cl/N3(DIB) device shows the highest *μ*_h_ of 6.75 × 10^−4^ cm^2^ V^−1^ s^−1^ and *μ*_e_ of 6.23 × 10^−4^ cm^2^ V^−1^ s^−1^, and a more balanced *μ*_h_/*μ*_e_ of 1.08 among the three systems, which also contributes to the observed high *J*_sc_ and FF outputs in the D18‐Cl/N3(DIB) device.^[^ ^15^ ^]^

In order to investigate the degree to which various charge recombination processes occur in all three OSC systems, *J*_sc_ versus light intensity (*P*_light_) curves were plotted in Figure 2b. The relationship between *J*_sc_ and *P*_light_ follows a power law: *J*_sc_ ∝ (*P*_light_)*^*α*^*; where *α* is an exponential factor that is indicative of the dominant charge recombination process. The closer *α* approaches unity, the weaker the bimolecular recombination processes occurring in the active layer of the device.^[^ ^16^ ^]^ Even though all three samples display similar *α* values, the D18‐Cl/N3(DIB) device still exhibits the highest value of 0.998 compared to that of D18‐Cl:N3 and D18‐Cl/N3 (*α* = 0.993 and 0.996, respectively), suggesting that the DIB additive induces less bimolecular recombination.

To gain further insight into the carrier dynamics process in device, transient photocurrent (TPC) and transient photovoltage (TPV) were measured. The carrier lifetimes were extracted from TPC and TPV decay curves by using mono‐exponential fits.^[^ ^17^ ^]^ As shown in Figure 2c, D18‐Cl/N3(DIB) devices show a shorter carrier lifetime (0.16 *μ*s) than those of D18‐Cl:N3 devices (0.22 *μ*s) and D18‐Cl/N3 devices (0.21 *μ*s), indicating more efficient carrier extraction. Note that this is in good agreement with the enhanced charge collection probability in D18‐Cl/N3(DIB) devices.^[^ ^9^, ^18^ ^]^ As exhibited in Figure 2d, the carrier lifetime of D18‐Cl:N3, D18‐Cl/N3, and D18‐Cl/N3(DIB) devices are 0.31, 0.42, and 0.54 *μ*s under one Sun illumination, respectively. The relatively longer carrier lifetime in D18‐Cl/N3(DIB) devices implies significantly reduced charge recombination.^[^ ^19^ ^]^ These results adequately account for the device performance difference among the three systems.

**Figure 3:**
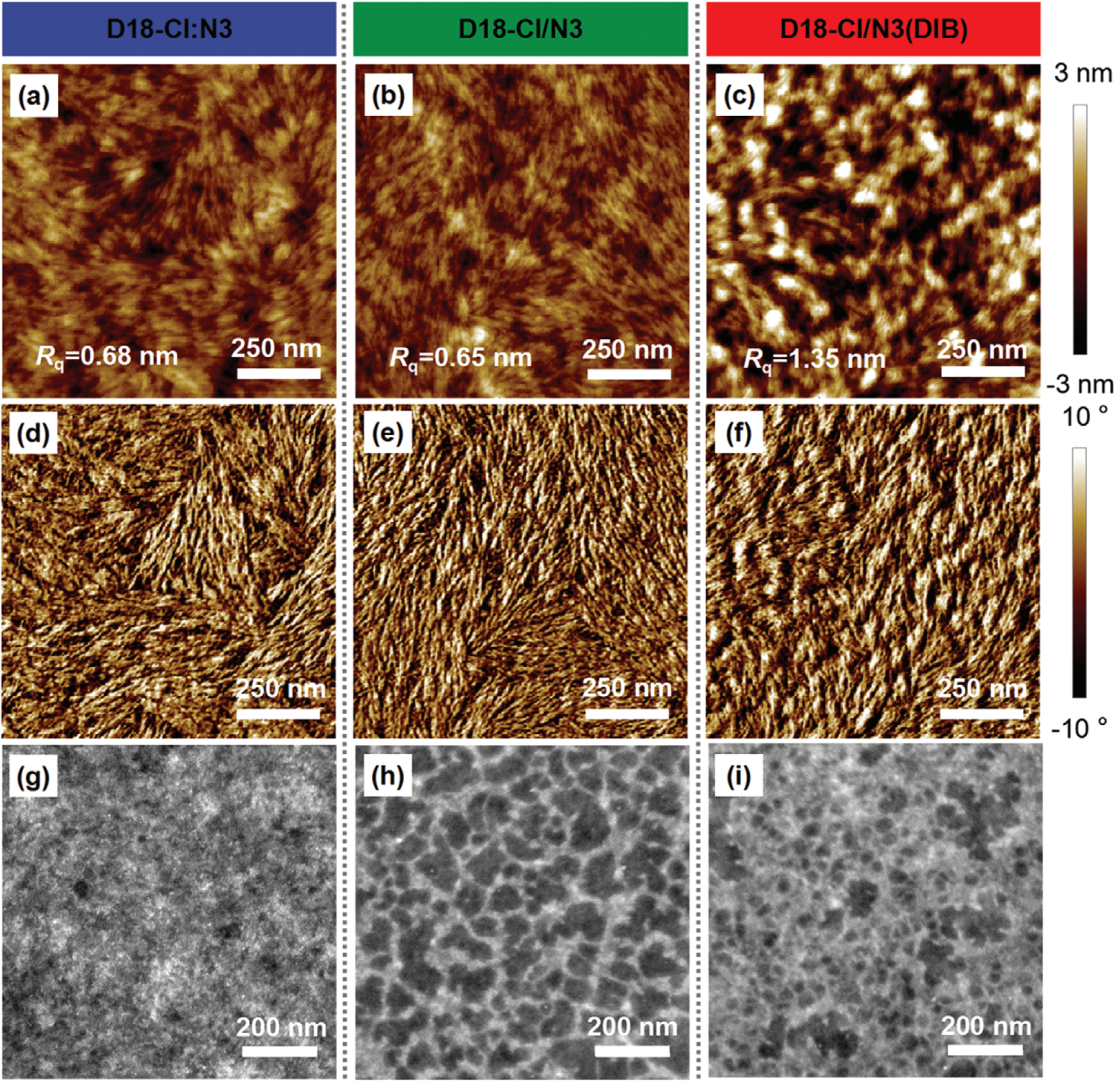
AFM height images of a) D18‐Cl:N3, b) D18‐Cl/N3, and c) D18‐Cl/N3(DIB) blend films. AFM phase images of d) D18‐Cl:N3, e) D18‐Cl/N3, and f) D18‐Cl/N3(DIB) blend films. TEM images of g) D18‐Cl:N3, h) D18‐Cl/N3, and i) D18‐Cl/N3(DIB) blend films.

To further probe the underlying morphological influence on device performance, atomic force microscopy (AFM) characterization was used to investigate the surface morphology of all three blend films. As shown in **Figure** **3**a–f, interpenetrating fiber networks were observed in both D18‐Cl:N3 and D18‐Cl/N3 blend films. This suggests that even though a SD method was employed, the acceptor N3 is able to permeate into the D18‐Cl fiber network and form a BHJ‐like structure in the D18‐Cl/N3 blend film. Once processed with the DIB additive, the surface of D18‐Cl/N3(DIB) becomes much rougher. Compared with D18‐Cl/N3 film, D18‐Cl/N3(DIB) film shows finer phase separation, which increases the interfacial areas of donor/acceptor domain and thus effectively promotes exciton dissociation probability.

In addition, Figure S5 (Supporting Information) are the AFM images for the neat films added with DIB. The root‐mean‐square roughness (*R*_q_) of neat D18‐Cl film is largely unaffected, while the *R*_q_ of neat N3 film is significantly increased by more than six times. These observations suggest that the volatile DIB additive is effective mainly on the acceptor material. It can effectively regulate the surface texture and self‐assembly of acceptor N3, delivering greatly enhanced molecular crystallinity.

Transmission electron microscopy (TEM) measurements were performed to further probe the bulk morphology of active layer. Thanks to the percolation behavior in the SD process, the D18‐Cl/N3 film shows well‐defined domains compared to that of D18‐Cl:N3 film (Figure 3g,h). Upon adding DIB, the D18‐Cl/N3(DIB) film exhibit more appropriate nanoscale phase separation (Figure 3i). Such a favorable morphology accounts for the observed excellent exciton dissociation and charge transport processes in the D18‐Cl/N3(DIB) devices.

To further investigate the molecular stacking and crystalline feature in neat and blend films, grazing‐incidence wide‐angle X‐ray scattering (GIWAXS) measurement was conducted. Figure S6a (Supporting Information) presents the 2D GIWAXS patterns of the neat D18‐Cl and N3 films. The corresponding line cuts in the in‐plane (IP) and out‐of‐plane (OOP) directions are shown in Figure S6b (Supporting Information). The neat D18‐Cl film shows a strong (010) *π*–*π* stacking peak in the OOP direction at *q* = 1.63 Å^−1^ and a (100) lamellar peak in the IP direction at *q* = 0.32 Å^−1^, suggesting its preferential face‐on orientation.^[^ ^20^ ^]^ The neat N3 film also adopts the dominant face‐on orientation with an obvious *π*–*π* stacking peak in the OOP direction at *q* = 1.73 Å^−1^ and a (100) lamellar peak in the IP direction at *q* = 0.30 Å^−1^.^[^ ^12^ ^]^

**Figure 4:**
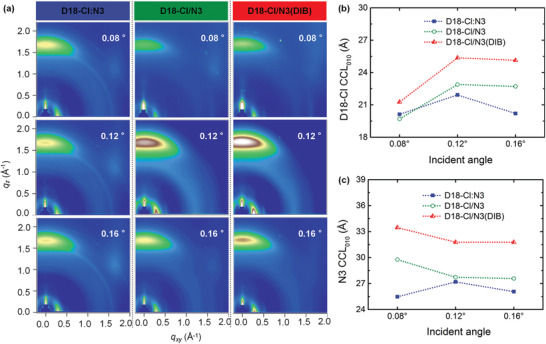
a) 2D GIWAXS patterns of D18‐Cl:N3, D18‐Cl/N3, and D18‐Cl/N3(DIB) blend films at different incident angles (0.08°, 0.12°, and 0.16°). The corresponding CCL_010_ values of b) D18‐Cl and c) N3 in three blend films at different incident angles.

To explore the vertical phase separation differences among the three blend systems, angle‐dependent GIWAXS measurement was performed with the incident angle varied from 0.08° to 0.16° (**Figure** **4**a and Figure S7, Supporting Information).^[^ ^17^, ^21^ ^]^ Apparently, all samples show a dominant face‐on orientation, which facilitates efficient charge transport process in the vertical direction.^[^ ^22^ ^]^ The corresponding 1D profiles were fitted to quantitatively evaluate the *π*–*π* stacking coherence lengths (CCL_010_) of donor and acceptor crystallites in the OOP direction (Figure 4b,c; Table S7, Supporting Information). For the D18‐Cl donor component in blends and at a low incidence angle of 0.08°, the D18‐Cl/N3 blend film possesses a slightly smaller D18‐Cl donor CCL_010_ value than that in D18‐Cl:N3 film. However, with the increase of incident angle, the CCL_010_ of D18‐Cl component in D18‐Cl/N3 blend film becomes larger than that in D18‐Cl:N3 blend film. Particularly, after volatile DIB treatment, the D18‐Cl/N3(DIB) blend film exhibits a large enhancement in CCL_010_ values for D18‐Cl component along the vertical phase. At the same time, for the N3 acceptor component in blends, the SD method‐processed blend films possess higher CCL_010_ values than that of OTD method along the vertical phase, especially on the top surface of film. As a result of the volatile DIB additive, the self‐assembly of acceptor N3 was significantly enhanced with the largest CCL_010_ value on the top surface of film. The synergistic regulation of donor/acceptor crystallinity along the entire vertical phase accounts for the observed enhancement in both charge transport and extraction process in the D18‐Cl/N3(DIB) devices. Collectively, the developed volatile solid additive‐assisted SD strategy demonstrates an effective morphology optimization strategy for high‐performance OSCs.

## 3 Conclusion

In summary, through utilization of the DIB volatile solid additive coupled with sequential deposition, we demonstrated a highly efficient OSC based on polymer donor D18‐Cl and acceptor N3. Meanwhile, angle‐dependent GIWAXS measurement was performed to reveal the vertical phase separation and molecular crystallinity of the active layer. The SD method delivers a BHJ‐like bicontinuous interpenetrating network with tunable phase distribution. Introducing volatile additive can further promote acceptor self‐assembly in blend, which helps to form finer phase separation with high crystallinity for efficient charge transport and collection. The synergistic effect results in a champion PCE of 18.42% with significantly enhanced *J*_sc_ of 27.18 mA cm^−2^ and FF of 78.8% in D18‐Cl/N3(DIB) devices. Our work provides a feasible pathway for simultaneous regulation of phase separation and crystallinity to achieve high‐performance OSCs.

## Conflict of Interest

The authors declare no conflict of interest.

## Supporting information

Supporting Information

## Data Availability

The data that support the findings of this study are available in the supplementary material of this article.
