# Supplementary material for: Volatile Solid Additive‐Assisted Sequential Deposition Enables 18.42% Efficiency in Organic Solar Cells
Source: Adv Sci (Weinh). 2022 Jan 24;9(9):2105347. doi: 10.1002/advs.202105347 (PMC8948555; doi:10.1002/advs.202105347)
Supplement: Supplementary file 1 — Supporting Information [file ADVS-9-2105347-s001.pdf]

ADVANCED SCIENCE
Open Access

Supporting Information

for *Adv. Sci.*, DOI 10.1002/advs.202105347

Volatile Solid Additive-Assisted Sequential Deposition Enables 18.42% Efficiency in Organic Solar Cells

Jianqiang Qin, Qianguang Yang, Jiyeon Oh, Shanshan Chen, George Omololu Odunmbaku, Nabonswendé Aïda Nadège Ouedraogo, Changduk Yang, Kuan Sun* and Shirong Lu**

WILEY-VCH

ADVANCED SCIENCE
Open Access

Supporting Information

for *Adv. Sci.*, DOI: 10.1002/advs.202105347

Volatile solid additive-assisted sequential deposition enables 18.42% efficiency in organic solar cells

Jianqiang Qin, Qianguang Yang, Jiyeon Oh, Shanshan Chen, George Omololu Odunmbaku, Nabonswendé Aïda Nadège Ouedraogo, Changduk Yang, Kuan Sun,* and Shirong Lu**

Supporting Information

Volatile solid additive-assisted sequential deposition enables 18.42% efficiency in organic solar cells

Jianqiang Qin, Qianguang Yang, Jiyeon Oh, Shanshan Chen, George Omololu Odunmbaku, Nabonswendé Aïda Nadège Ouedraogo, Changduk Yang, Kuan Sun,* and Shirong Lu**

J. Qin, Prof. S. Chen, Dr. G. O. Odunmbaku, Dr. N. A. N. Ouedraogo, Prof. K. Sun
MOE Key Laboratory of Low-grade Energy Utilization Technologies and Systems, School of Energy & Power Engineering, Chongqing University, Chongqing 400044, P. R. China
E-mail: shanshanchen@cqu.edu.cn(S. Chen); kuan.sun@cqu.edu.cn(K. Sun)

J. Qin, Q. Yang, Prof. S. Lu
Organic Semiconductor Research Center, Chongqing Institute of Green and Intelligent Technology, Chinese Academy of Sciences, Chongqing 400714, P. R. China
E-mail: lushirong@cigit.ac.cn(S. Lu)

J. Oh, Prof. C. Yang
Department of Energy Engineering, School of Energy and Chemical Engineering, Perovtronics Research Center, Low Dimensional Carbon Materials Center, Ulsan National Institute of Science and Technology (UNIST), Ulsan 44919, Republic of Korea

Experimental Section

1. Materials

The polymer donor D18-Cl was purchased from eFlexPV Co. The acceptor N3 was synthesized according to the previous report.[1] PDIN was purchased from Solarmer Material Inc.

2. Device fabrication and measurement

All the OSCs were fabricated with a conventional structure of ITO/PEDOT:PSS/active layer/PDIN/Ag. The ITO substrates (15 Ω sq^{-1}) were sequentially cleaned and sonicated with detergent, deionized water, acetone, and isopropyl alcohol. Then, ITO substrates were treated with UV-ozone for 15 min. Subsequently, a 30 nm PEDOT:PSS film was deposited on the ITO substrate with a speed of 4000 rpm for 30 s and then baked at 150 ℃ for 10 min before being transferred into N_2-filled glove box. For the BHJ active layer, the D18-Cl:N3 blend was dissolved in chloroform (CF) with a D:A ratio of 1:1.4 and a concentration of 16 mg mL^{-1}. The D18-Cl:N3 blend film was spin-coated on the PEDOT:PSS film at 4000 rpm for 30 s. For the sequential deposition (SD) active layer, the donor D18-Cl and acceptor N3 were dissolved into CF with a concentration of 6.5 and 7.5 mg mL^{-1}, respectively. Firstly, the D18-Cl was deposited on PEDOT:PSS film as front layer at 4000 rpm for 30 s and then the N3 solution was spin-coated on the top of D18-Cl film at 3000 rpm for 30 s. Before casting the acceptor N3, solid additive DIB was dissolved into N3 chloroform solution. The active layers were thermally annealed at 90 ℃ for 5 min to remove the solid additive. Subsequently, a thin PDIN (2 mg mL^{-1} in methanol with 0.3 vol% acetic acid) layer was spin-coated on the top of active layer. Finally, a 100 nm Ag electrode was thermally deposited under 2×10^{-6} Torr. The effective area of device was 10 mm^2. The *J-V* curves of devices were measured by Keithley 2400 under AM 1.5G (100 mW cm^{-2}) generated from a solar simulator. The illumination intensity was determined by a silicon photodiode calibrated by the National Renewable Energy Laboratory (NREL). The external quantum efficiency (EQE) spectra were performed by using QE-R equipment system (Enli Tech).

3. Ultraviolet-Visible (UV-Vis) absorption spectra and Fourier transform infrared (FT-IR) spectra

UV-Vis absorption spectra were measured by using a Lambda 365 UV/Vis spectrophotometer (PerkinElmer). FT-IR measurement was performed by using a FT-IR spectrometer (PerkinElmer).

4. SCLC measurement

The carrier (hole or electron) mobility was measured via space-charge-limited current (SCLC) method. The hole-only devices were fabricated with architecture of ITO/PEDOT:PSS/active layer/MoO_3/Ag, and the electron-only devices were fabricated with configuration of ITO/ZnO/Phen-NaDPO/active layer/Phen-NaDPO/Ag.[2] The *J*-*V* curves were measured by Keithley 2400 in the dark. The mobility was obtained by fitting the dark current with the following equation:

$$J = \frac{9}{8}\varepsilon_0\varepsilon_r\mu\frac{V^2}{L^3}$$

where *J* is the current density, ε_0 is the permittivity of the vacuum, ε_r is the dielectric constant of material, μ is zero-field mobility (electron mobility μ_e and hole mobility μ_h), *V* is the effective voltage ($V = V_{appl} - V_{bi}$, where V_{appl} and V_{bi} are the applied voltage and built-in potential, respectively), *L* is the film thickness. The mobility can be obtained from the $J^{1/2}$-*V* plot.[3]

5. Morphology characterization

Atomic force microscope (AFM) measurements were conducted on a Dimension Edge (Bruker) by using in tapping mode. Transmission electron microscope (TEM) measurements were performed on a Talos F200S instrument. The grazing-incidence wide-angle X-ray scattering (GIWAXS) measurements were carried out at PLS-II 9A beamline of the Pohang Accelerator Laboratory in Korea. The samples were prepared on Si substrates using the identical conditions with the device fabrication. The X-ray beam energy was set to 11.5 keV. The incident angle of X-ray beam was varied from 0.08 to 0.16 °. The crystal coherence length (CCL) was calculated by using the Scherrer equation:[4]

$$CCL = \frac{2\pi K}{\Delta q}$$

Where *K* is the shape factor ($K = 0.9$), and Δq is the full-width at half-maximum (FWHM) of the diffraction peak.

6. Transient photocurrent (TPC) and transient photovoltage (TPV) measurements

TPC measurements were performed under short-circuit condition in the dark. TPV measurements were acquired at open-circuit condition under one sun illumination from a white light-emitting diode. The output signals were recorded on a keysight oscilloscope.[5]

Supporting Figures

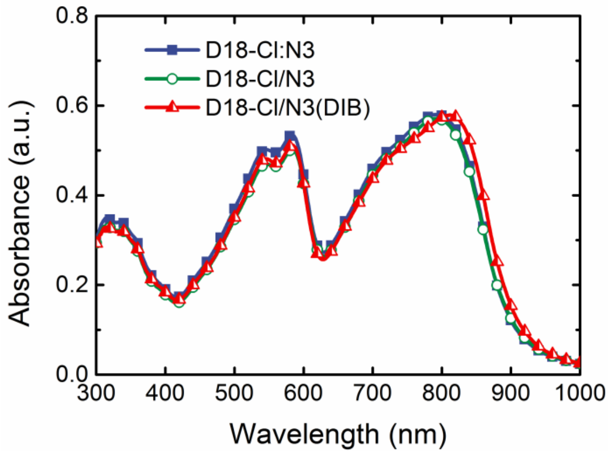

Figure S1 Absorption spectra of D18-Cl:N3, D18-Cl/N3, and D18-Cl/N3(DIB) blend films.

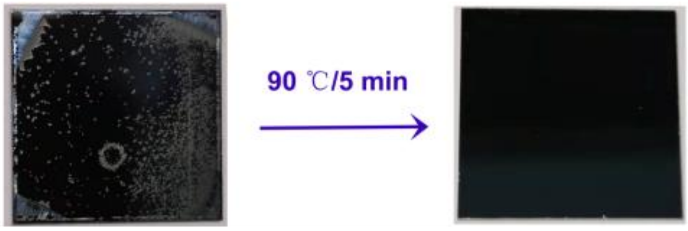

Figure S2 The images of DIB film coated on the silicon wafer before and after 90 ℃ thermal annealing for 5 min.

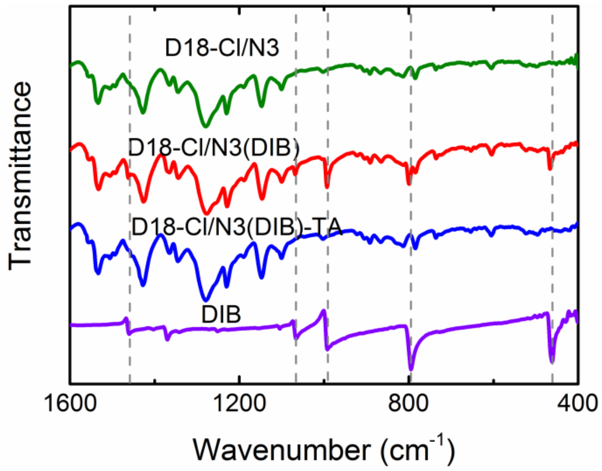

Figure S3 FT-IR spectra of DIB, D18-Cl/N3, and D18-Cl/N3(DIB) without and with TA treatment at 90 ℃ for 5 min.

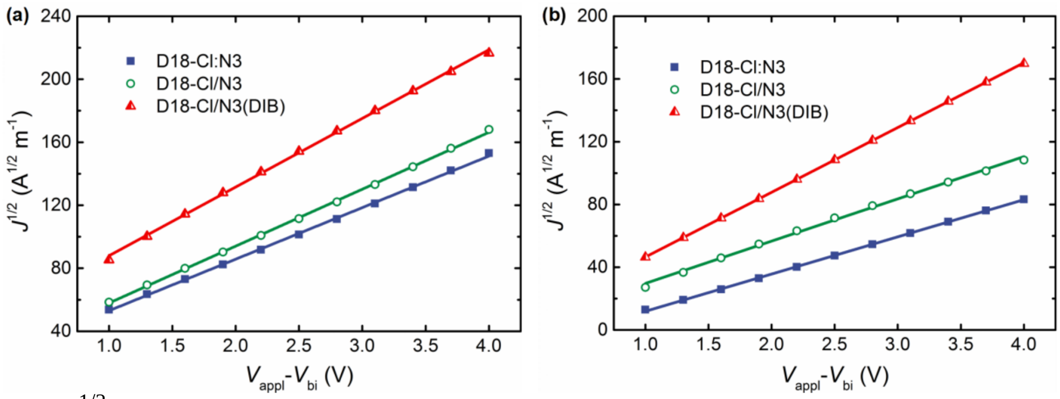

Figure S4 $J^{1/2}$-V plots (a) for hole-only devices and (b) for electron-only devices.

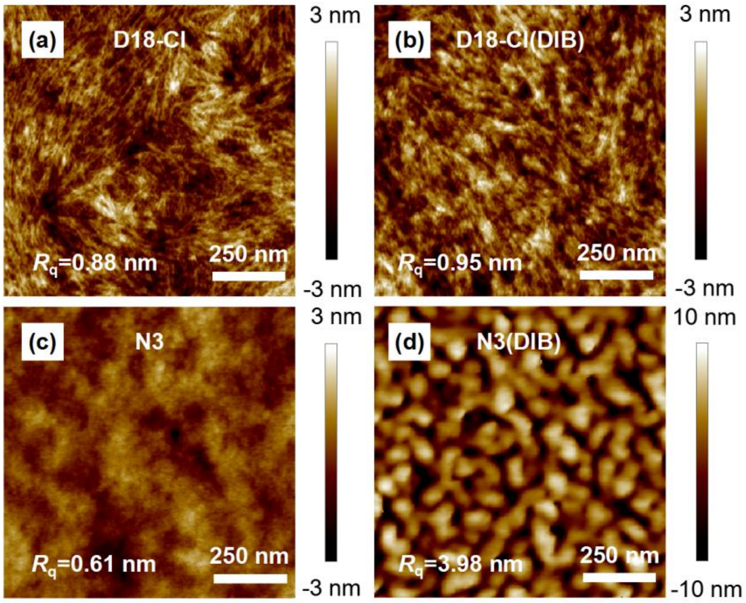

Figure S5 AFM height images of (a) D18-Cl, (b) D18-Cl(DIB), (c) N3, and (d) N3(DIB) neat films.

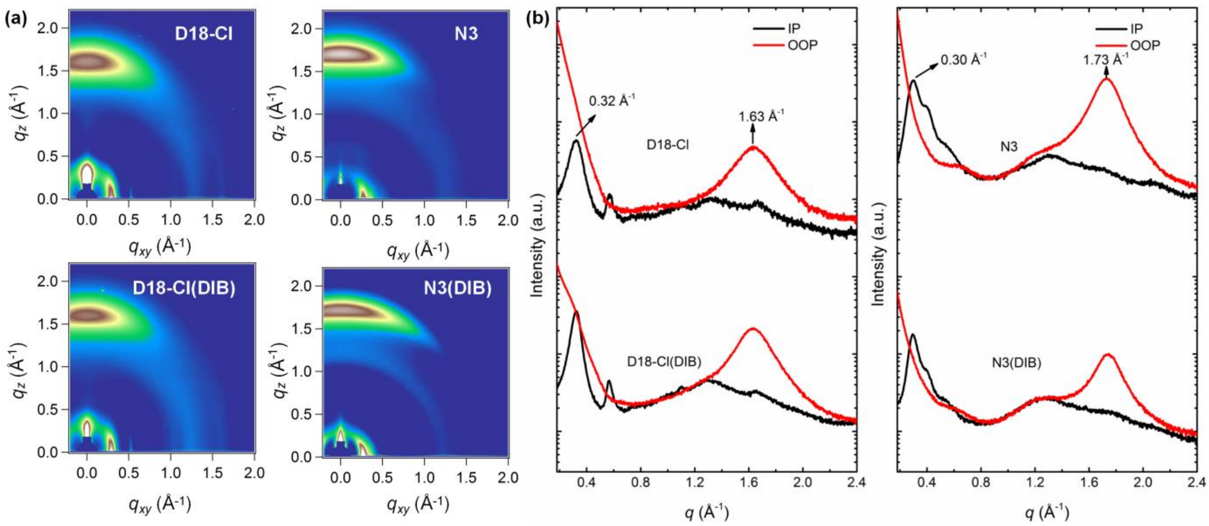

Figure S6 (a) 2D GIWAXS patterns and (b) the corresponding in-plane (IP) and out-of-plane (OOP) line cuts of D18-Cl, D18-Cl(DIB), N3, and N3(DIB) neat films.

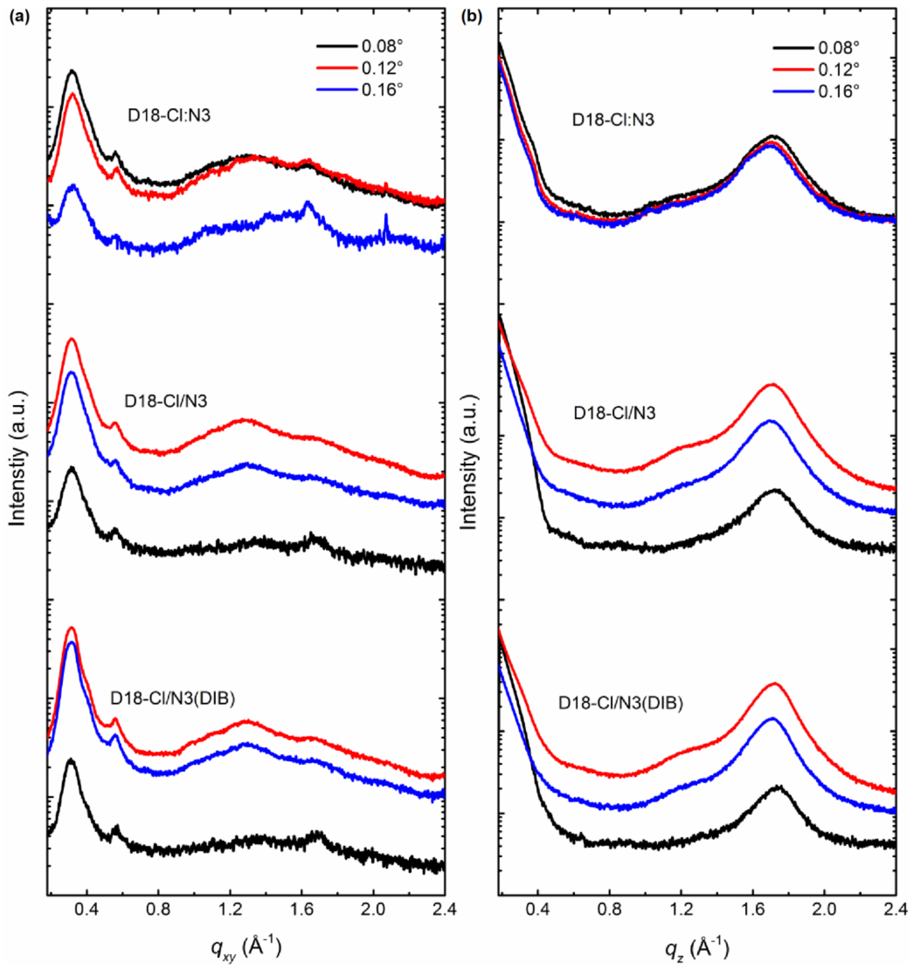

Figure S7 (a) In-plane and (b) out-of-plane line cuts of D18-Cl:N3, D18-Cl/N3, and D18-Cl/N3(DIB) blend films with different incident angles (0.08 °, 0.12 °, and 0.16 °).

Supporting Tables

Table S1 Optimization of thickness for donor in SD OSCs.

Donor/Active layer [nm]	V_{oc} [V]	J_{sc} [mA cm^{-2}]	FF [%]	PCE[a] [%]
80/118	0.872	26.35	76.1	17.47 (17.08 ± 0.35)
65/104	0.870	26.27	76.9	17.58 (17.35 ± 0.21)
57/98	0.870	25.99	77.1	17.43 (17.02 ± 0.35)
47/90	0.867	25.69	77.8	17.32 (16.93 ± 0.31)

[a]Average values and standard deviation were obtained from 16 individual devices.

Table S2 Optimization of thickness for acceptor in SD OSCs.

Donor/Active layer [nm]	V_{oc} [V]	J_{sc} [mA cm^{-2}]	FF [%]	PCE[a] [%]
65/135	0.867	26.58	76.4	17.60 (17.19 ± 0.32)
65/110	0.873	26.74	76.0	17.74 (17.50 ± 0.17)
65/95	0.868	26.37	76.2	17.44 (17.17 ± 0.19)

[a]Average values and standard deviation were obtained from 16 individual devices.

Table S3 Optimization of DIB content in N3 for SD OSCs.

DIB [mg mL^{-1}]	V_{oc} [V]	J_{sc} [mA cm^{-2}]	FF [%]	PCE[a] [%]
0	0.873	26.74	76.0	17.74 (17.50 ± 0.17)
5	0.864	26.84	78.0	18.07 (17.68 ± 0.26)
10	0.860	27.18	78.8	18.42 (18.20 ± 0.15)
15	0.861	27.04	78.1	18.19 (17.83 ± 0.20)
20	0.861	26.56	77.2	17.64 (17.33 ± 0.25)

[a]Average values and standard deviation were obtained from 16 individual devices.

Table S4 Summary of recently reported high-performance binary SD OSCs.

Donor	Acceptor	V_{oc} [V]	J_{sc} [mA cm^{-2}]	FF [%]	PCE [%]	Reference
PBDB-TFS1	IT-4F	0.90	20.3	71	13.0	[6]
PM6	Y6	0.834	25.90	75.68	16.35	[7]
PT2	Y6	0.83	26.7	74.4	16.5	[8]
PM6	Y6-BO	0.847	26.2	77.5	17.2	[9]
D18	N3	0.834	27.79	75.61	17.52	[10]
PM6	L8-BO	0.89	26.11	80.6	18.74	[11]

Table S5 Exciton dissociation and charge collection probability of the devices.

Active layer	η_{diss}	η_{coll}
D18-Cl:N3	97.4%	86.6%
D18-Cl/N3	97.7%	87.6%
D18-Cl/N3(DIB)	98.1%	90.7%

Table S6 Hole and electron mobility.

Films	μ_h [cm^2 V^{-1} s^{-1}]	μ_e [cm^2 V^{-1} s^{-1}]	μ_h/μ_e
D18-Cl:N3	3.48×10^{-4}	1.88×10^{-4}	1.85
D18-Cl/N3	4.24×10^{-4}	2.66×10^{-4}	1.59
D18-Cl/N3(DIB)	6.75×10^{-4}	6.23×10^{-4}	1.08

Table S7 Detailed GIWAXS data of (010) peak in OOP direction.

Films	Incident angle	Component	q [Å^{-1}]	d-spacing [Å]	FWHM [Å^{-1}]	CCL [Å]
D18-Cl:N3	0.08 °	D18-Cl	1.648	3.81	0.281	20.12
		N3	1.735	3.62	0.222	25.47
	0.12 °	D18-Cl	1.632	3.85	0.258	21.92
		N3	1.733	3.63	0.208	27.19
	0.16 °	D18-Cl	1.620	3.88	0.280	20.20
		N3	1.715	3.66	0.217	26.06

D18-Cl/N3	0.08 °	D18-Cl	1.661	3.78	0.287	19.70
		N3	1.742	3.61	0.190	29.76
	0.12 °	D18-Cl	1.662	3.78	0.247	22.89
		N3	1.735	3.62	0.204	27.72
	0.16 °	D18-Cl	1.646	3.82	0.249	22.71
		N3	1.724	3.64	0.205	27.58
D18-Cl/N3(DIB)	0.08 °	D18-Cl	1.662	3.78	0.266	21.26
		N3	1.751	3.59	0.169	33.46
	0.12 °	D18-Cl	1.654	3.80	0.223	25.36
		N3	1.740	3.61	0.178	31.77
	0.16 °	D18-Cl	1.641	3.83	0.225	25.13
		N3	1.727	3.64	0.178	31.77

References

[1] K. Jiang, Q. Wei, J. Y. L. Lai, Z. Peng, H. K. Kim, J. Yuan, L. Ye, H. Ade, Y. Zou, H. Yan, *Joule* **2019**, *3,* 3020.

[2] H. Chen, D. Hu, Q. Yang, J. Gao, J. Fu, K. Yang, H. He, S. Chen, Z. Kan, T. Duan, C. Yang, J. Ouyang, Z. Xiao, K. Sun, S. Lu, *Joule* **2019**, *3,* 3034.

[3] Q. Liu, Y. Jiang, K. Jin, J. Qin, J. Xu, W. Li, J. Xiong, J. Liu, Z. Xiao, K. Sun, S. Yang, X. Zhang, L. Ding, *Sci. Bull.* **2020**, *65*, 272.

[4] J. Song, M. Zhang, M. Yuan, Y. Qian, Y. Sun, F. Liu, *Small Methods* **2018**, *2*, 1700229.

[5] J. Lv, H. Tang, J. Huang, C. Yan, K. Liu, Q. Yang, D. Hu, R. Singh, J. Lee, S. Lu, G. Li, Z. Kan, *Energy Environ. Sci.* **2021**, *14*, 3044.

[6] Y. Cui, S. Zhang, N. Liang, J. Kong, C. Yang, H. Yao, L. Ma, J. Hou, *Adv. Mater.* **2018**, *30*, 1802499.

[7] R. Sun, Q. Wu, J. Guo, T. Wang, Y. Wu, B. Qiu, Z. Luo, W. Yang, Z. Hu, J. Guo, M. Shi, C. Yang, F. Huang, Y. Li, J. Min, *Joule* **2020**, *4*, 407.

[8] K. Weng, L. Ye, L. Zhu, J. Xu, J. Zhou, X. Feng, G. Lu, S. Tan, F. Liu, Y. Sun, *Nat. Commun.* **2020**, *11*, 2855.

[9] H. Fu, W. Gao, Y. Li, F. Lin, X. Wu, J. H. Son, J. Luo, H. Y. Woo, Z. Zhu, A. K. Y. Jen, *Small Methods* **2020**, *4*, 2000687.

[10] Y. Wei, J. Yu, L. Qin, H. Chen, X. Wu, Z. Wei, X. Zhang, Z. Xiao, L. Ding, F. Gao, H. Huang, *Energy Environ. Sci.* **2021**, *14*, 2314.

[11] X. Xu, L. Yu, H. Meng, L. Dai, H. Yan, R. Li, Q. Peng, *Adv. Funct. Mater.*, DOI: 10.1002/adfm.202108797.
